# Social Functioning as a Mediator between Developmental Language Disorder (DLD) and Emotional Problems in Adolescents

**DOI:** 10.3390/ijerph18031221

**Published:** 2021-01-29

**Authors:** Claire L. Forrest, Jenny L. Gibson, Michelle C. St Clair

**Affiliations:** 1 Department of Psychology and Human Development, UCL Institute of Education, London WC1H 0AA, UK; Claire.forrest@ucl.ac.uk; 2 Faculty of Education, University of Cambridge, Cambridge CB2 8PQ, UK; jlg53@cam.ac.uk; 3 Department of Psychology, University of Bath, Bath BA2 7AY, UK

**Keywords:** developmental language disorder, adolescents, peer problems, emotional problems, mediation, developmental pathways

## Abstract

Adolescents with Developmental Language Disorder (DLD) are at risk for increased feelings of anxiety and depression compared to their typically developing (TD) peers. However, the underlying pathways involved in this relationship are unclear. In this initial study of the ‘social mediation hypothesis’, we examine social functioning as a mediator of emotional problems in a cross-sectional sample of adolescents with DLD and age- and sex-matched controls. Preliminary data from twenty-six participants with DLD and 27 participants with typical language development (TLD, 11–17 years) were compared on self- and parent-reported measures of social functioning and emotional outcomes. There was little evidence of group differences in self-reported social functioning and emotional outcomes, but parent-report of SDQ Peer Problems and Emotional Problems in the DLD group was significantly higher than in the TLD group. Parent-reported peer problems mediated parent-reported emotional problems, accounting for 69% of the relationship between DLD status and emotional problems. Parents of adolescents with DLD, but not adolescents themselves, report significantly higher peer and emotional problems compared to TLD peers. The hypotheses generated from these novel data suggest further investigation into adolescents’ perceptions of socioemotional difficulties and friendships should be examined.

## 1. Introduction

Adolescence is a time of increased social and emotional difficulties in general [1], but individuals with developmental language disorder (DLD) may struggle even more than their typically developing (TD) peers [2]. DLD is a difficulty with receptive and/or expressive language that cannot be accounted for by any other neurodevelopmental disorders, hearing impairments, or global intellectual difficulties, and it affects approximately 7–8% of the population [3,4]. Studies have shown that adolescents with a history of DLD are at increased risk for peer problems [5], anxiety [6], and depression [7] and that these difficulties persist throughout the life-span [8]. However, it is still unclear what mechanisms are responsible for these additional socioemotional difficulties [9], and previous research has focused on children [10]. Given adolescence is a critical period for the onset of later psychiatric problems [11], it is crucial to examine potential pathways to these increased emotional problems for a better understanding of the relationship that may lead to better interventions and care.

### 1.1. DLD and Emotional Difficulties

There are conflicting findings about the extent to which language ability influences emotional outcomes in children and young people with DLD. These discrepancies relate to the rater (e.g., parent vs. teacher) [12,13], or to the specific aspect of language that is measured (e.g., receptive vs. expressive) [2,14]. Moreover, some studies have found no direct link between language ability and emotional outcomes, either concurrently [7] or longitudinally [15], suggesting that other factors may be involved. Wadman et al. [16], for instance, found that peer problems linked to DLD predicted depression at age 16. There is a wide range of evidence suggesting that peer problems and victimization lead to mental health difficulties in the general population [17,18], and indeed that social support can act as a protective factor against depression [19]; therefore, this area warrants further investigation. Due to the increase in social problems in adolescents with DLD [2,15], we propose that social functioning may mediate the relationship between DLD status and emotional problems in this population.

### 1.2. DLD and Social Functioning

Social functioning is defined in this paper as quality of friendships and social activities, assessing both positive and negative aspects to provide a comprehensive picture of adolescents’ abilities in this domain. Language is integral to social functioning, and the effect is visible from a young age, with children aged 18–35 months who were identified as “late-talkers” demonstrating lower social competence than their age-matched peers [20]. Therefore, it is logical to assume that children and young people with DLD will have more difficulties with making friends and learning social skills. Indeed, teachers rate children with language difficulties as more withdrawn than their typically developing classmates [21,22], and observational studies demonstrate that children with DLD struggle to initiate conversation with their peers [23]. In turn, these limited social interactions could exacerbate social problems by providing fewer opportunities to learn and practice social skills, leading to a depleted social repertoire from which to draw upon in future interactions [24]. Children with DLD have demonstrated difficulty with social skills such as conflict resolution [25,26] or hiding emotions to protect others’ feelings [27,28].

These impaired social skills could have negative consequences for individuals’ social standing, as evidenced by children with DLD receiving more “dislike” ratings than their typically developing peers [29,30]. Children and young people with DLD are consistently rated by different informants (e.g., parent, teacher, and self) as experiencing more peer problems than others their age [5,12,31]. Furthermore, children with DLD have been found to experience higher rates of victimization compared to their typically developing peers [32,33] and compared to those with other developmental disorders such as Attention Deficit Hyperactivity Disorder [34]. In contrast, Lindsay, Dockrell, and Mackie [35] found similar rates of victimization in children with DLD, their TD peers, and those with other learning disabilities. Nevertheless, there is strong evidence that children and young people with DLD experience more difficulties with social functioning than their peers.

### 1.3. The Emotional Impact of Social Difficulties

The overall lower social competence experienced by children and adolescents with DLD may have detrimental effects on their mental health. For instance, findings from the general population demonstrate that poor social competence at 4 years of age predicts later internalizing and externalizing problems at 10 and 14 years of age [36], and there is evidence to suggest that peer relations predict concurrent depressive symptoms in adolescents with DLD [16]. Indeed, some adolescents with DLD report higher feelings of social stress [37] and social phobia [38], highlighting the psychological impact of poor social competence. In order to unravel this relationship further, researchers have examined potential mediating factors that may explain the association between DLD and poor socioemotional outcomes. For example, shyness has been found to mediate the relationship between language ability and self-esteem in adolescents and young adults with DLD [39,40], highlighting the link between sociability and mental health.

### 1.4. Current Study

**Figure 1:**
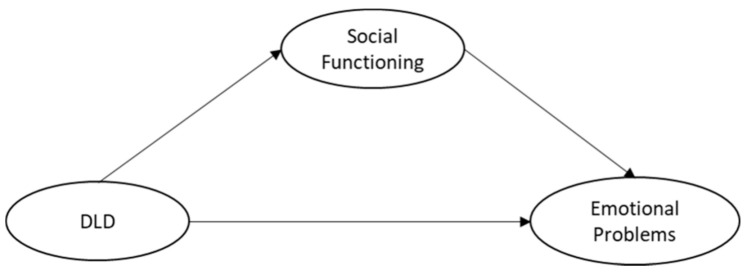
The social mediation hypothesis. This model illustrates the proposed mediating effect of social functioning on the relationship between Developmental Language Disorder (DLD) and emotional problems.

Previous studies have demonstrated an elevated rate of social and emotional problems in children with DLD; however, the research on adolescents and potential mechanisms to explain the relationship is lacking. The latter is considered particularly important for helping identify targets for preventive interventions. The current study puts forward the “social mediation hypothesis” (Figure 1) and presents preliminary data to test this model. Friendships become more complex as children enter adolescence, moving away from the play-based activities of childhood and focusing more on talking, particularly for girls [41]. Additionally, peer relationships become more important during this time, helping adolescents build a stronger sense of identity and providing emotional support [42]. Added to the fact that adolescence is a critical time for the onset of later psychiatric disorders [11], this is a key period to study. There is a vast literature demonstrating the protective effect of friendships on mental health in the general population [19,43]. Adolescents with DLD may be at a disadvantage compared to their typically developing peers if they have not had the same opportunities to form strong friendships. Therefore, poor emotional outcomes in adolescents with DLD may be the result of poor social functioning, not the direct effect of poor language ability.

Data were analyzed at the group level in line with the evidence that suggests there is no linear relationship between language ability and severity of socioemotional problems in children with DLD [21,44]. Furthermore, as outlined above, adolescents with DLD may differ from their peers in their development of social skills and friendships due to fewer opportunities to socialize. Therefore, it may be group status (i.e., DLD or TLD) that has an effect on socioemotional outcomes, and not the extent of the language difficulty itself. The mediating effect of peer problems on emotional outcomes has already been shown in a population study of young people considered to be at risk of DLD [45]. This effect was found across time (middle childhood to early adolescence) and across informants (teacher-rated and parent-rated). While the sample size of the current study is much smaller, the use of a cross-sectional, matched sample expands on previous findings by using a range of parent-report and self-report measures of social and emotional functioning to provide a more comprehensive account of the difficulties that adolescents with DLD may face. It is expected that adolescents with DLD will experience higher levels of emotional difficulties than their age- and sex-matched peers, as reported by their parents and themselves. In addition, it is hypothesized that the young people with DLD will report fewer successful experiences of social functioning than the control group, as evidenced by higher rates of victimization and lower perceived social support. Furthermore, poorer social functioning is expected to mediate the relationship between language status and emotional outcomes.

## 2. Materials and Methods

### 2.1. Recruitment

**Figure 2:**
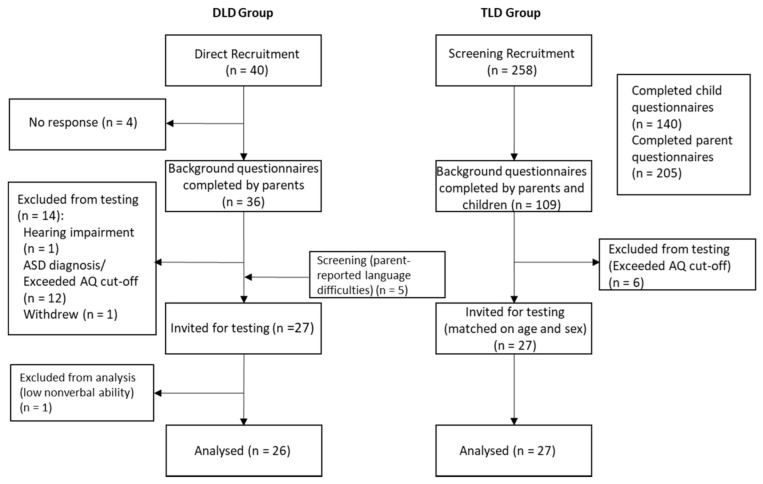
Flowchart of participant recruitment. AQ, Autism Quotient; ASD, Autism Spectrum Disorder; DLD, Developmental Language Disorder; TLD, Typical Language Development.

There were two recruitment streams for the study; participants with a diagnosis of DLD were recruited through direct referrals, whereas a screening procedure identified the TLD comparison group (see Figure 2). Participants were included in the DLD group if they were aged 11–18 years old, native English speakers, and had a history of DLD. Those who had no language difficulties were individually matched on age (within six months) and sex to form the TLD comparison group. Any participants with a history of language difficulties identified through the screening procedure were included in the DLD group (n = 5). Exclusion criteria consisted of parent-report of hearing impairments, intellectual disabilities, and diagnoses of autism spectrum conditions. The Autism Quotient (AQ) [46,47] was also included as an exclusionary measure. Eighteen participants were excluded due to exceeding the cut-off on the AQ (n = 12 from DLD group, n = 6 from TLD group).

Participants were recruited between Spring 2016 and Spring 2018, either through referrals from a local speech and language therapy service and from Special Educational Needs Coordinators (SENCos) within schools, or from flyers posted locally or on social media. All participants attended mainstream schools, although three participants in the DLD group were recruited from a specialized language unit within a mainstream school.

Forty individuals with a diagnosis of DLD were initially identified as potential participants. Four participants did not respond to invitation emails. One participant was excluded due to a hearing impairment, twelve participants were excluded due to a diagnosis of autism or exceeding the cut-off on the AQ, and one participant withdrew from the study.

Screening packs, consisting of background questionnaires for parents and self-report measures for adolescents, were sent to 258 participants to recruit the TLD comparison group. Of the 109 that were completed by both parent and participant, six were excluded due to exceeding the AQ cut-off and five were included in the DLD group due to parent report of language difficulties, including one participant with a low score on the self-report measure (CC-SR—see methods).

Thus, twenty-seven participants were invited to the testing stage as part of the DLD group, with twenty-seven matched controls forming the TLD group. One further participant was excluded from the DLD group after scoring more than 2 SDs below the mean on the nonverbal measure. This resulted in a total sample of 26 adolescents with DLD and 27 TLD participants matched on age and sex. Further details of the sample characteristics are provided in Table 1.

### 2.2. Participants

Table 1 shows the demographics of the sample. The total sample had an average age of 13 years and 6 months (*SD* = 16.45 months) and approximately 36% were female (n = 19). English was the only language spoken at home the majority of the time, although one participant in the DLD group spoke German as well, and two participants in the TLD group also spoke German and Spanish. As expected, the DLD group were significantly more delayed in speech and language development compared to the TLD group. They were also more delayed in reaching early self-help milestones compared to the TLD group. Level of parental education significantly differed between the two groups, with more parents in the TLD group completing postgraduate studies compared to the DLD group. Additionally, the DLD group had a significantly lower socioeconomic status as measured by the Income Deprivation Affecting Children Index (IDACI) Rank Z-score. In the current sample, the IDACI Rank Z-score ranged from −2.36 to 2.54 in the DLD group and from −0.26 to 2.30 in the TLD group, with an overall group mean of 0.58 (*SD* = 0.83). The DLD group consisted of individuals from significantly more deprived areas than the TLD group.

### 2.3. Measures

#### 2.3.1. Parent-Report

Parents completed a background questionnaire designed for the current study, consisting of twenty-four questions regarding the child’s early development and language spoken at home. Questions included the speed at which developmental milestones in language, motor skills, and self-help were met and whether the child had any learning difficulties (suspected or diagnosed), including specific difficulties in language development. Family history of learning difficulties and mental health difficulties (suspected or diagnosed) was also asked, with the latter providing information about parental psychological distress. Postcode information was also gathered, which provided a measure of socioeconomic status based on the Income Deprivation Affecting Children Index (IDACI) Rank. The IDACI Rank is based on the percentage of children living in families that are income deprived in Lower-layer Super Output Areas (LSOAs) across England, where 1 = most deprived neighborhood and 32,844 = least deprived neighborhood. School postcodes were used when home postcodes were missing (n = 5) and two participants in the DLD group had neither information available. Based on recommendations by Bishop [48] the IDACI Ranks were transformed into z-scores for ease of comparison.

##### Parent-Report of Socioemotional Abilities

Socioemotional outcomes were measured by the parent report form of the Strengths and Difficulties Questionnaire [49]. The SDQ consists of 25 items that form five scales (Peer Problems; Emotional Problems; Hyperactivity; Conduct Problems and Prosocial scale), the first four of which are totaled to produce the Total Difficulties score. Each item is rated on a scale of Not True, Somewhat True and Certainly True with scores of 0, 1, or 2 assigned to each rating, respectively. The SDQ is a well-established measure with a test-retest reliability of 0.85 [50] and acceptable reliability [51]. The scales of interest were the Emotional Problems and Peer Problems subscales, each consisting of five items. Total scores for each subscale range from 0–10 with a higher score indicating more problems.

##### Parent-Report of Autism

The adolescent version of the Autism Spectrum Quotient [46] was administered to parents of children aged 12–15 years old. The AQ consists of 50 items referring to the domains of social skills (e.g., “S/he prefers to do things with others rather than on his/her own”); attention switching (e.g., “S/he prefers to do things the same way over and over again”); attention to detail (e.g., “S/he often notices small sounds when others do not”); communication (e.g., “Other people frequently tell her/him that what s/he has said is impolite, even though s/he thinks it is polite”) and imagination (e.g., “S/he finds making up stories easy”). Items are rated as “Definitely agree”, “Slightly agree”, “Slightly disagree” or “Definitely disagree”, and responses that endorse autistic-like behaviors are scored 1 point. A sum score of 30 or more on the parent-report is classified as a cut-off for ASD symptoms and was used as an exclusion criterion for participants in the current study.

#### 2.3.2. Adolescent-Report for Screening Sample Only

##### Adolescent-Report of Language Abilities

The Communication Checklist Self-Report [52] was completed by the participants recruited into the TLD group. This questionnaire consists of 70 questions about communication abilities. The participant rated the items on a scale of 0—Less than once a week (or never); 1—About once a week; 2—Once or twice a day or 3—Several times a day (or all the time). These items form three composite scales. The Structural Language composite describes aspects of language such as grammar and meaning. For example, “I mix up ‘he’, “she”, “it” and “they” and “I use short sentences”. The Pragmatic Skills composite contains items relating to language use in social contexts. For instance, “People tell me I talk too much” and “I give detailed information when a more general comment would be fine”. Finally, the Social Engagement composite is comprised of items regarding nonverbal communication and social functioning. For example, “I feel anxious when I am with other people” and “I find it hard to know when people are upset or annoyed”. Positive items are reverse scored and a scaled score lower than 5 on the Structural Language composite and greater than 7 on the Pragmatic Skills composite is indicative of DLD (M = 10, SD = 3). Internal consistency for each of the composites is greater than 0.85 [52].

The adult version of the AQ (Baron [47] was completed by participants aged 16 years or over. The adult version consists of the same 50 items and five domains as the adolescent version [46] and is rated on the same scale; however, the cut-off criterion is slightly higher with a sum score of 32 or more on the self-report considered to be indicative of ASD symptoms and was used as an exclusion criterion for participants in the current study.

#### 2.3.3. Adolescent Assessment

##### Language Assessment

Two subtests from the Clinical Evaluation Language Fundamentals—4 [53] were administered to assess language ability. The Recalling Sentences subtest requires participants to listen to sentences of increasing length and complexity and repeat verbatim, providing a measure of expressive language. The Word Classes subtest requires participants to pick two words out of a list of four that are best matched and provide a definition for how the two words are similar. This subtest provides a measure of receptive language abilities in the current study. Both subtests have an excellent rating of reliability [53].

##### Nonverbal Ability

The Block Design subtest from the Wechsler Intelligence Scale for Children [54] was administered to provide a measure of nonverbal ability. This task requires participants to use 3D blocks to recreate 2D patterns of increasing complexity. Block Design is a measure of spatial awareness and contributes to fluid reasoning. Participants scoring more than 2 SDs below the mean (M = 10, SD = 3) were excluded from the study.

#### 2.3.4. Adolescent Self-Report of Social and Emotional Outcomes

##### Friendships

Questions about friendships (‘Do you have any best friends?’ ‘How often do you argue with your friends?’); victimization (How often do other children hurt you or pick on you on purpose?) and bullying (‘How often do you hurt or pick on other children on purpose?’) were adapted from the “Your Friends” section of the fifth wave of the Millennium Cohort Study [55]. The question ‘How often do you argue with your friends?’ and the victimization and bullying questions were rated on scales of Most days, About once a week, About once a month, Every few months, Less often, and Never. For ease of analysis, these scales were reclassified into new categories of Yes or No.

##### Social Support

The Perceived Social Support—Friendship [56] consists of 20 statements about friendship quality and measures how well participants believe their support needs are met by their relationships with friends. Participants answer Yes, No or Don’t Know depending on how true they feel each statement is. Ratings of ‘Yes’ are scored 1 while ratings of “No” and “Don’t Know” receive scores of 0. The PSS-Fr has good internal consistency (α = 0.88), and items are summed to create a total score ranging from 0-20, with a higher score indicating higher perceived social support [56].

##### Mental Well-Being

The Warwick Edinburgh Mental Well-being Scale [57] consists of 14 items focusing on positive attributes, and previous research indicates this is both a good measure of mental wellbeing as well as mental health difficulties [58]. Participants rate statements on a scale of 1 None of the time–5 All of the time, according to how they felt in the past two weeks. Scores range from 14–70, with a higher score reflecting a better state of mental wellbeing. Internal consistency is good or excellent, ranging from α = 0.89 to 0.91 based on university student and population samples [57].

##### Anxiety

The Revised Children’s Manifest Anxiety Scale [59] consists of 28 statements regarding feelings of anxiety. The current study uses an adapted four-category version from [58] where participants are asked to rate each item according to how they have been feeling in the past two weeks on a scale of ‘Never’, ‘Sometimes’, ‘Mostly’ and ‘Always’. Answers were assigned scores of 0–4, respectively, resulting in a total score ranging from 0–84. A higher score indicates more feelings of anxiety. Internal consistency for the adapted version in this study was excellent (α = 0.94).

##### Depressive Symptoms

The Mood and Feelings Questionnaire [60] consists of 33 statements that measure depressive thoughts and feelings over the last two weeks. The current study uses an adapted four-category version obtained from St Clair et al. (2017), where participants respond to each item how often they have felt or acted in this way over the past two weeks on a scale of ‘Never’, ‘Sometimes’, ‘Mostly’ and ‘Always’. Scores of 0–4 are assigned to each rating, resulting in a total score ranging from 0–99, with higher scores indicating more depressive symptoms. Internal consistency for the adapted version in this study was excellent (α = 0.94).

### 2.4. Procedure

Ethical approval was granted by the University of Bath Psychology Ethics Committee (REF: 15–245). Questionnaires were hosted online (Qualtrics.com) or administered via paper copy. Informed consent and assent were obtained from parents/guardians and participants (see S1). Parents/guardians in the DLD group completed the consent, background questionnaire, AQ and SDQ online or returned the forms in a freepost envelope. Participants from the DLD group were then invited to the assessment stage either at the University, their school or their home. Participants who were recruited through schools gave assent and were screened with the CC-SR while their parents gave consent and completed the SDQ and AQ. In order to reduce time and encourage screening completion a shortened version of the background questionnaire was administered to parents/guardians during this stage. Again, the assessment stage was completed wherever was convenient for the participant. Parents/guardians recruited through screening completed online consent forms for the assessment stage, along with the remaining background questionnaire. Participants completed online assent forms at the beginning of the assessment. Participants were administered the two language tasks, the four socioemotional questionnaires, and the Block Design task. Each questionnaire item was read aloud to every participant. The entire procedure lasted 1.5 h in total (including other tasks that address separate research questions that will be presented elsewhere). In the event of a participant reporting suicidal ideation on the MFQ a safety protocol was followed, which involved a structured interview to ascertain how often the participant experienced those feelings and whether or not they were likely to act upon them. The participant’s parent was notified of these findings and was sent a referral letter for their GP. Participants received £15 on completion of the assessment stage and any travel expenses were reimbursed. As an incentive to complete the screening process participants recruited through this manner were entered into a prize draw to win a £50 shopping voucher. Brief reports of individuals’ results were sent to parents/guardians, and findings from the overall study were shared with parents/guardians in the form of a newsletter.

### 2.5. Statistical Analysis

SPSS 25 [61] was used to analyze the data. Following tests for assumptions, chi squares, linear regression, and ordered logistic regression were used to analyze group differences in the demographic variables. Socioeconomic status (measured by IDACI Rank Z-score) was included as a covariate in all analyses due to the significant group difference. Group differences in cognitive and language measures were analyzed using MANOVA. Chi-squares were used to compare group differences in the dichotomous variables of friendship and victimization. Group differences in wellbeing (WEMWBS) and social support (PSS-Fr) were analyzed using linear regression, while the variables of anxiety and depression (RCMAS and MFQ) were transformed due to significant skew before regression analysis. Group differences in the SDQ subscales of Peer Problems and Emotional Problems were analyzed using negative binomial regression due to the most frequent response being zero. The mediating effect of social functioning was analyzed using the PROCESS procedure [62], which tests the significance of indirect effects, and bootstrapping with case resampling was used to generate confidence intervals. Both of these methods are viewed as a more robust measure of mediation than the traditional [63] method. In order to test the effect of mediation, only social functioning variables, which had a significant group difference (path a) were entered and only emotional variables that had a significant group difference (path c) were entered into the model.

## 3. Results

### 3.1. Cognitive and Language Measures

A MANOVA illustrated that there was a significant group difference on all cognitive and language measures, F(3, 46) = 21.86, *p* < 0.001, Wilk’s Λ = 0.41, *ηp^2^* = 0.59. Table 2 shows the DLD group scored significantly lower than the TLD group on the measures of spatial reasoning, expressive language and receptive language. However, it should be noted that the overall mean score for the DLD group was still within the normal range for the Block Design subtest (*M* = 10, *SD* = 3).

### 3.2. Social and Emotional Outcomes

Table 3 shows the social outcomes in each group from self- and parent-report. There was a significant group difference in membership of social clubs, with participants in the TLD group more likely to attend social clubs than participants in the DLD group (*X*^2^ = 8.27, *p* < 0.01, Phi = −0.40). Surprisingly, there were no significant group differences in report of best friends, but adolescents in the TLD group reported arguing more with their friends than the DLD group (*X*^2^ = 5.64, *p* < 0.05, Phi = −0.33). More participants in the DLD group reported being bullied than the TLD group, whereas the TLD group reported more incidences of bullying others than the DLD group but these differences were not statistically significant (*p* = 0.328 and *p* = 0.140, respectively). The DLD group and TLD group reported receiving similar levels of social support from their friends. As expected, the DLD group received significantly higher ratings than the TLD group on the parent-rated SDQ subscale of Peer Problems.

Table 4 demonstrates self-report and parent-report of emotional outcomes in each group. Self-reports of anxiety and depression were higher in the DLD group compared to the TLD group, but these differences were not significant. Similarly, the DLD group reported lower feelings of mental wellbeing than the TLD group but this was not a statistically significant difference. However, as expected, the DLD group received significantly higher ratings than the TLD group on the parent-rated SDQ subscale of Emotional Problems.

### 3.3. Social Functioning as a Mediator

**Figure 3:**
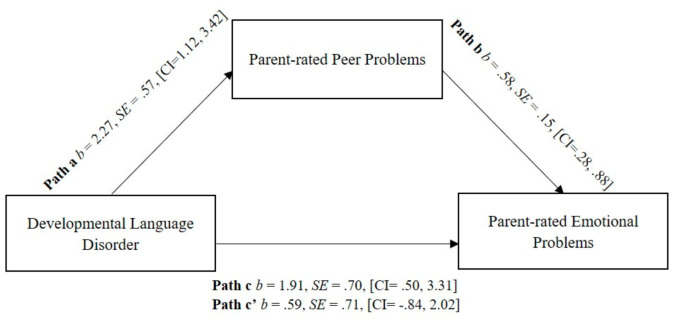
Regression coefficients for the relationship between Developmental Language Disorder (DLD) and parent-rated emotional problems as mediated by parent-rated peer problems.

The association between DLD and parent-reported emotional problems was mediated by parent-reported peer problems. Figure 3 illustrates that DLD group status predicted parent-rated peer problems, t(51) = 3.96, *p* < 0.001 and these peer problems were significantly related to emotional problems, t(50) = 3.82, *p* < 0.001. The effect of DLD group status on emotional problems was not significant after controlling for peer problems (t(50) = 0.59, *p* = 0.409), consistent with mediation. The proportion of the total effect of DLD status on emotional problems that operated indirectly through peer problems was 69.1% using bootstrapping, *b* = 1.32, bootSE = 0.44, [bootCI = 0.52, 2.28]. A moderated mediation to test the effect of arguing with friends on this model was not significant, as there was no interaction effect t(49) = −0.57, *p* = 0.570.

## 4. Discussion

The current paper examined social functioning differences as a potential mediator of emotional problems in adolescents with DLD. By using a mediation analysis in an adolescent sample matched on age and sex, we aimed to further the research into the pathways involved in the relationship between language difficulties and poor emotional outcomes in this population. Furthermore, by using self-report and parent-report of social and emotional problems, we aimed to provide a comprehensive account of the additional difficulties that adolescents with DLD may face as self-reports in this area are not as frequently examined as parent or teacher reports [31]. It is important to note that these data are generated from a small sample and therefore findings should be interpreted with caution.

Contrary to expectations, the DLD group reported significantly fewer problems with their friends than the TLD group, with just over half reporting that they never argue with their friends. This could reflect a less nuanced understanding of friendships or the poorer verbal abilities of the DLD group, which limit the extent to which they can successfully engage in disagreements with their peers [25,26]. Additionally, a smaller proportion of the DLD group attended social clubs compared to the TLD group. This could reflect a greater difficulty in facilitating group interactions and socializing with others that is seen in children and young people with DLD [23,37,38]. Although not significant, there were expected trends in victimization as over half (61.5%) of adolescents in the DLD group reported being bullied compared to less than half (48.1%) in the TLD group. The DLD group reported that they bullied others less frequently than the TLD group, but this difference was also not significant. More adolescents in the DLD group reported the presence of a best friend than their TLD peers. Although this finding is not significant the direction is surprising given the literature on higher peer problems in adolescents with DLD. It may be that reports of a ‘best friend’ in the current study indicate one close friend, but not a larger group of friends. Indeed, many participants in the TLD group, but not the DLD group, asked for clarification on this item as they could not choose only one best friend out of their friendship group. Despite some differences in the level of conflict and victimization, both groups reported similar rates of social support from their peers. These findings are consistent with Wadman et al. [37], who found no significant difference between adolescents with and without DLD in perceived social competence or acceptance amongst peers. Moreover, longitudinal research has highlighted that not all adolescents with DLD have peer problems, with approximately a third of the participants followed-up from the MLS cohort reporting no difficulties or having peer problems resolved by adolescence [5].

While these non-significant results are encouraging in that adolescents with DLD do not appear to be experiencing higher rates of social difficulties compared to their TLD peers, it is important to consider alternative interpretations given that the majority of previous literature suggests adolescents with DLD are at greater risk for social difficulties. Admittedly, both Wadman et al. [37] and the current study used small samples of 28 or 26 adolescents with DLD compared to the larger samples from the Manchester Language Study (MLS) cohort, which found significant group differences in self-report of social difficulties [14,31]. However, it should also be noted that the MLS papers used the SDQ self-report of peer problems whereas the current study and Wadman et al. [37] included positive measures of social competence and support. Specifically, the SDQ taps into problems with peer relationships, such as playing alone or being bullied by others, while the PSS-Fr provides more insight into the quality of friendships and how much emotional support is shared. This difference in measurement of social functioning may explain the difference in findings. However, the measures of negative social outcomes in the current study, such as victimization and arguing with friends, were not significantly higher in the DLD group, which contradicts findings from the literature [32,33,34].

In contrast to the lack of significant problems reported by the adolescents with DLD, their parents perceived them to experience significantly higher rates of peer problems. This could reflect the higher levels of victimization that the DLD group reported. While comparison between raters was not the aim of this study, the discrepancy between parent and self-report is an interesting finding. Administering the self-report SDQ would allow for a direct comparison, but the measures used in the current study were chosen to reflect a wider range of social functioning. Alternatively, the difference between raters could be due the adolescents with DLD having a less sophisticated understanding of friendship, while their parents are more aware of their child’s difficulties with their peers. Again, this raises the question of the factors involved in the relationship. Adolescents with DLD may have difficulty conceptualizing social competence due to fewer successful experiences, leading them to perceive fewer problems than their parents. Recent research has examined the effect of social cognition on social outcomes in adolescents with DLD [64]. but this has not been extended to emotional outcomes. Additionally, future studies could employ a qualitative design to provide a deeper insight into how adolescents with DLD view their social functioning.

There were no significant group differences in self-reported emotional problems, which contrasts previous findings of increased feelings of anxiety and depression in adolescents with DLD compared to TLD peers [7]. However, the direction of results was as expected, with the DLD group reporting higher mean scores than the TLD group for negative outcomes such as feelings of anxiety and depression and lower mean scores for the positive outcome of mental wellbeing. The current study used the same standardized self-report measures of emotional outcomes as previous literature; therefore, the difference in these findings compared to previous studies is likely due to the smaller sample size in the current study and the potential for reduced power to find significant differences. However, the reduced sample size does not explain the significantly higher ratings of parent-reported emotional problems in the DLD group compared to the TLD group, which are similar to previous studies [7,12]. Again, adolescents may be lacking in the metacognitive skills necessary for this self-reflection, while the difficulties may be more salient for parents. Or, indeed, there is potential for parental concerns about language abilities to bias their reporting in other domains. Qualitative studies investigating adolescents’ attitudes towards DLD and understanding of friendship and how this impacts them emotionally could help further unravel the relationship with associated socioemotional difficulties.

When parent-rated peer problems were entered into a mediation model the relationship between DLD and parent-rated emotional problems was non-significant, with peer problems accounting for 69.1% of the relationship. In other words, the increased emotional problems found in the DLD group can be mostly explained by increased peer problems. This is consistent with previous research that found peer problems predicted depressive feelings in adolescents with DLD [16,45]. When emotional problems were entered as the mediator and peer problems as the outcome, the mediation effect was reduced to approximately 32.8% of the relationship, suggesting that there is a stronger effect of peer problems predicting emotional problems than vice versa. This is in line with the body of literature from the general adolescent population examining victimization as a causal factor in psychopathology [17,65] and findings that show friendship support is a positive predictor of adaptive psychosocial functioning [66].

This study extends the literature by examining the mediating effect of peer problems on emotional problems in an adolescent DLD sample. By employing a cross-sectional design and using a typical-language comparison group matched on age and sex, we were able to directly compare between adolescents with and without a language difficulty, in contrast to previous studies that have relied on comparison to normative means [2]. Using a clinical sample also enriches previous findings based on a cohort sample of individuals at risk of DLD [45]. Additionally, social and emotional measures were collected from adolescents themselves, as well as parents. This allowed for first-person report of the difficulties that this population are said to experience, which is often lacking in studies centered on younger children. The focus on adolescents in the current study is useful for determining the pathways involved in socioemotional difficulties during a time when peer relationships become more influential and when peer problems and emotional problems are likely to increase. The scales used to measure social and emotional functioning were validated measures that are frequently cited in the literature. In addition, we included questions about friendships and social activities to measure social functioning, providing more detailed information about the ‘lived experiences’ of adolescents. Furthermore, the PSS-Fr provides a measure of positive aspects of friendship, while the WEMWBS provides a general overview of mental wellbeing. It is important to provide a report of strengths as well as difficulties that adolescents with DLD may face in order to ascertain any protective factors.

Moreover, the current study provided further details on how the relationship between language disorder and emotional problems may manifest. By examining peer problems as a mediator, we have proposed that the increased rates of emotional difficulties are a result of the difficulties with friendships that adolescents with DLD experience, and not simply a result of their language difficulties. This preliminary study gives insight into the pathways involved in the poor mental health outcomes in this population and provides potential avenues for intervention, such as strategies to improve social relationships that in turn will alleviate emotional difficulties. Another key finding for clinicians to consider is the different perspectives of socioemotional functioning in the DLD population from both adolescents and their parents. With any discrepant self/other reports it is often difficult to determine which report is the most reliable; however, considering the well-established increased mental health difficulties in individuals with DLD [6,7,9], we can tentatively conclude that the adolescents in this study may be under-reporting or simply lack awareness of their difficulties. Supporting adolescents to understand and express their feelings may go some way to reduce this discrepancy between raters, as well as help to strengthen peer relationships by encouraging adolescents to monitor and manage their emotions during social interactions for more successful exchanges. A further area for clinicians to focus on may be supporting adolescents with understanding social cues, given the suggestion that adolescents may be misinterpreting their social interactions. Again, as demonstrated by the social mediation model, this may help to reduce emotional difficulties as well.

The current study is not without its limitations. The sample size is relatively small and therefore may not be an accurate representation of the population. As a result, the study may be lacking in statistical power, and the conclusions drawn are at risk of being based on type II error. Nevertheless, this initial study has generated hypotheses that may be tested in future studies with a larger sample. Additionally, with the exception of the PSS-Fr and Peer Problems subscale from the SDQ, the social functioning questions were taken from surveys administered in the Millennium Cohort Study (MCS) and not from standardized scales. However, the TLD participants were matched on age and sex, so comparisons could be made between young people with DLD and their typically developing peers at a group level. There was a significant group difference in nonverbal ability, which could explain some of the differences in social and emotional outcomes, but this was difficult to control for.

## 5. Conclusions

In this initial study of the social mediation model, parent-reported peer problems were found to mediate the relationship between DLD status and parent-reported emotional problems, suggesting that a difficulty interacting with others explains over half the variance in emotional problems experienced by adolescents with DLD. However, the DLD group do not report any significant social or emotional difficulties themselves when compared to their TLD peers. These preliminary findings allow for hypothesis refining. For example, it could be that adolescents with DLD do not perceive the same difficulties as their parents due to a deficit in social cognition abilities. Given the long-term effects of emotional difficulties, the proposed area of social cognition warrants further investigation in order to uncover the mechanism driving the difference in emotional outcomes between adolescents with DLD and their typically developing peers.

## Figures and Tables

**Table 1:** Demographics of sample.

	DLD (n = 26)	TLD (n = 27)	Total (n = 53)	DLD vs. TLD
Mean age in yrs; mths (SD months)	13;6 (16.06)	13;6 (17.13)	13;6 (16.45)	--
Female % (n)	34.6 (10)	37 (9)	35.8 (19)	*X*^2^ 0.03
Mean IDACI Z-score (SD) ^a^	0.28 (0.91)	0.84 (0.68)	0.58 (0.83)	−0.56^(−1.01, −0.11) **
Language spoken				*X*^2^ 0.31
English only %	96.2	92.6	94.3	
English plus other %	3.8	7.4	5.7	
Motor development				0.49^(0.15, 1.59)
Delayed %	26.9	3.7	15.1	
Typical %	53.8	81.5	67.9	
Fast %	19.2	14.8	17	
Speech and language development				0.05^(0.01, 0.21) ***
Delayed %	69.2	7.4	37.7	
Typical %	26.9	70.4	49.1	
Fast %	3.8	22.2	13.2	
Self-help development				0.17^(0.05, 0.63) **
Delayed %	46.2	3.7	24.5	
Typical %	42.3	85.2	64.2	
Fast %	11.5	11.1	11.3	
Biological parents				*X*^2^ 2.25
Yes %	92.0	100.0	96.2	
No—adopted %	8.0	0	3.8	
Parental marital status				3.85^(0.89, 16.55)
Married %	68.0	88.9	78.8	
Separated %	16.0	7.4	11.5	
Divorced %	16.0	3.7	9.6	
Parental psychological distress ^b^				*X*^2^ 0.01
Yes %	16.0	14.8	15.4	
No %	84.0	85.2	84.6	
Parental education				0.19^(0.06, 0.58) **
Secondary school %	44.0	18.5	30.8	
Diploma %	12.0	0	5.8	
Undergraduate degree %	36.0	44.4	40.4	
Postgraduate degree %	8.0	37.0	23.1	

DLD, Developmental Language Disorder; TLD, Typical Language Development; IDACI, Income Deprivation Affecting Children Index. Statistics are *b* coefficients or odds ratio where marked ^ (95% confidence interval) and chi square were marked *X*^2^. ^a^ n = 51. A lower score denotes higher levels of deprivation. ^b^ As measured by endorsement of suspected or diagnosed mental health difficulties on background questionnaire. **. *p* < 0.01, ***. *p* < 0.001.

**Table 2:** Comparison of mean (SD) scaled scores from cognitive and language tasks by Developmental Language Disorder (DLD) group and Typical Language Developed (TLD) group.

	DLD (n = 26)	TLD (n = 27)	F	*p*	*η_p_* ^2^
Spatial Reasoning ^a^	8.15 (2.84)	11.89 (2.47)	19.64	<0.001	0.29
Expressive Language ^b^	4.92 (2.93)	9.93 (2.96)	29.21	<0.001	0.38
Receptive Language ^c^	6.04 (2.92)	13.00 (2.39)	67.27	<0.001	0.58

MANOVA controlling for IDACI z-score. ^a^ Wechsler Intelligence Scale for Children—fourth edition UK (WISC-IV) Block Design subtest. ^b^ Clinical Evaluation of Language Fundamentals—fourth edition UK (CELF-4) Recalling Sentences subtest. ^c^ CELF-4 Word Classes-Receptive subtest.

**Table 3:** Mean (and standard deviation) ratings of social functioning outcomes from self-report and parent-report.

	DLD	TLD	DLD vs. TLD	Effect Size
Self-report				
Best Friend			*X*^2^ 3.20, n.s.	
Yes (%)	84.6	63.0		-
No (%)	15.4	37.0		
Argue with friends			*X*^2^ 5.64 *	Φ − 0.33
Yes (%)	46.2	77.8		
No (%)	53.8	22.2		
Member of social clubs			*X*^2^ 8.27 **	Φ − 0.40
Yes (%)	65.4	96.3		
No (%)	34.6	3.7		
Victim of bullying			*X*^2^ 0.96, n.s.	-
Yes (%)	61.5	48.1		
No (%)	38.5	51.9		
Bully others			*X*^2^ 2.18, n.s.	-
Yes (%)	7.7	22.2		
No (%)	92.3	77.8		
Perceived Social Support—Friendship Scale (PSS-Fr)	11.15 (3.71)	11.22 (2.65)	−0.13[−1.98, 1.71] n.s.	-
Parent-report				
SDQ Peer Problems	3.31 (2.53)	1.04 (1.56)	1.11[0.43, 1.80] **	*d* −1.09[−1.67, −0.51] ***

Statistics are chi-squares or *b* coefficients [95% confidence interval], controlling for IDACI Rank Z-score. SDQ Peer Problems = Strengths and Difficulties Questionnaire (SDQ) Peer Problems subscale. *. *p* < 0.05, **. *p* < 0.01, ***. *p* < 0.001.

**Table 4:** Mean (and standard deviation) ratings of mental health outcomes from self-report and parent-report.

	DLD	TLD	DLD vs. TLD	Cohen’s d [95% CI]
Self-report				
Warwick Edinburgh Mental Wellbeing Scale (WEMWBS)	51.96 (9.28)	54.22 (6.52)	−1.27[−7.61, 2.19] n.s.	0.28[−0.26, 0.82]
Revised Children’s Manifest Anxiety Scale (RCMAS)	20.54 (15.77)	12.93 (10.53)	0.87[−0.15, 1.89] n.s.	−0.46[−1.0, −0.09]
Moods and Feelings Questionnaire (MFQ)	14.65 (14.14)	9.00 (9.46)	0.48[−0.62, 1.58] n.s.	−0.27[−0.81, 0.27]
Parent-report				
SDQ Emotional Problems	3.54 (2.97)	1.63 (2.06)	0.83[0.15, 1.50] *	−0.75[−1.30, −0.20] **

Statistics are *b* coefficients [95% confidence interval], controlling for IDACI Rank Z-score. Regressions for RCMAS and MFQ are performed on transformed data. *. *p* < 0.05, **. *p* < 0.01.

## Data Availability

The data that support the findings of this study are available from the corresponding author, M.C.S.C, upon reasonable request.

## Supplementary Materials

The following are available online at https://www.mdpi.com/1660-4601/18/3/1221/s1, Text S1: Informed consent.
