# Supplementary material for: Social Functioning as a Mediator between Developmental Language Disorder (DLD) and Emotional Problems in Adolescents
Source: Int J Environ Res Public Health. 2021 Jan 29;18(3):1221. doi: 10.3390/ijerph18031221 (PMC7908163; doi:10.3390/ijerph18031221)
Supplement: Supplementary file 1 [file ijerph-18-01221-s001.pdf]

S1: Informed consent

Bath Language Study

Department of Psychology
UNIVERSITY OF BATH
Bath BA2 7AY · United Kingdom

Telephone +44 (0)1225 383843
Facsimile +44 (0)1225 386752
Email
psychology-enquiries@bath.ac.uk
www.bath.ac.uk/psychology

Dear parent/caregiver,

After reading this project summary please complete the consent form and questionnaires.

Summary of the project:

- Your child is invited to take part in a project that aims to understand the link between language ability, social skills and well-being.
- You will be asked to fill in a short questionnaire regarding any family history of learning disabilities or mental health problems as well as additional information regarding your child's developmental history. You will also be asked to fill in a questionnaire about your child's strengths and difficulties.
- Your child will be administered tasks to assess their receptive and expressive language skills as well as general cognitive ability. They will then complete computer tasks to assess their social functioning and answer questionnaires about their well-being and friendships.

Contact: If you require any further information or have any questions about this study, please do not hesitate to contact us at bathlangstudy@bath.ac.uk. Alternatively, our supervisor Dr Michelle St Clair can be contacted at: Address: Dr Michelle St Clair, Department of Psychology, University of Bath, BA2 7AY.
Phone: 01225 384393
E-mail: m.c.st.clair@bath.ac.uk

Declaration of parental consent. Please read each statement carefully and tick if you agree.

	Please tick
I confirm that I have read and understood the project summary and have had an opportunity to ask any questions	○
I confirm that I understand my child’s information will be kept strictly confidential	○
I confirm that I consent to having an audio recording made of my child’s responses to selected tasks. I understand that this is for research purposes only	○
I understand that participation is completely voluntary and my child and myself are free to withdraw from the project at any time, without giving a reason	○
I agree that my anonymous data and my child's anonymous data can be used in this project and future research projects	○
I agree to give consent for my child to take part in the project	○
I agree to consent to take part in the project	○

Child's name:

____

Child's gender:

○ Male

○ Female

Child's date of birth (DD/MM/YYYY):

____

Parent/Guardian's relationship to child:

- ○ Mother
- ○ Father
- ○ Stepmother
- ○ Stepfather
- ○ Other (Please specify) ____

Email address:

____

Phone number:

____
